# Diagnostic flow analysis of tuberous sclerosis complex in Japan: a retrospective claims database study

**DOI:** 10.1186/s13023-024-03460-y

**Published:** 2024-12-01

**Authors:** Tohru Okanishi, Ikuo Fujimori, Mariko Yamada, Takumi Tajima, Mari Wataya-Kaneda, Kuniaki Seyama, Takashi Hatano

**Affiliations:** 1 Division of Child Neurology, Brain and Neurosciences, Faculty of Medicine, Tottori University, Yonago, Japan; 2 Novartis Pharma K.K, 1-23-1 Toranomon, Minato-ku, Tokyo, 105-6333 Japan; 3 Division of Real-World Evidence, JMDC Inc., Tokyo, Japan; 4 Department of Neurocutaneous Medicine, Division of Health Science, Graduate School of Medicine, Osaka University, Osaka, Japan; 5 Department of Dermatology, Graduate School of Medicine, Osaka University, Osaka, Japan; 6 Division of Respiratory Medicine, Faculty of Medicine and Graduate School of Medicine, Juntendo University, Tokyo, Japan; 7 Department of Urology, Seirei Yokohama Hospital, Yokohama, Japan

**Keywords:** Tuberous sclerosis complex, Epidemiology, Japan, Delayed diagnosis, Real-world evidence, Health insurance claims, Multidisciplinary care system

## Abstract

**Background:**

Tuberous sclerosis complex (TSC) is a rare autosomal dominant genetic disorder that affects multiple organs. However, precise diagnosis is challenging owing to the lack of truly pathognomonic symptoms. This retrospective observational study aimed to explore the real-world diagnostic flow of Japanese patients with TSC by examining time to diagnosis (TTD) from the onset of each TSC-related manifestation to TSC diagnosis and the role of TSC clinic in timely diagnosis, using data from a health insurance database.

**Methods:**

Analyses were performed using data derived from the JMDC Claims Database between January 2005 and December 2020. Patients with at least 1 confirmed diagnosis of TSC were stratified into 2 cohorts: Cohort 1 included cases diagnosed after 2 years of age, and Cohort 2 included cases diagnosed before 2 years of age. The primary endpoint was TTD in Cohorts 1 and 2. Secondary endpoints were the incidence of each manifestation in Cohort 1 and the incidence and risk ratios of TSC-unrelated symptoms in Cohort 2.

**Results:**

Cohorts 1 and 2 included 106 and 42 patients, respectively. In Cohort 1, patients with a renal tumor diagnosis as a primary TSC-related manifestation had the longest TTD with a wide range (median: 23 months to up to 91 months); patients with non-specific TSC-related manifestations such as brain tumor/intraventricular tumor, epilepsy, or intellectual disabilities also experienced a delay in TTD. In patients with TSC who developed epilepsy, those attending facilities with a TSC clinic were diagnosed with TSC more quickly than those attending facilities without a TSC clinic (median: 11.5 and 19.0 months, respectively; *p* = 0.0379). Epilepsy was the manifestation with the highest incidence (29.2%) among Cohort 1 patients, while cardiac rhabdomyoma had the highest incidence (54.8%) among Cohort 2 patients. Dry skin was the most common TSC-unrelated symptom in Cohort 2, with a 1.7-fold higher incidence rate than that in controls (N = 619,936).

**Conclusion:**

Japanese patients with renal lesions as a primary TSC-related manifestation had the longest delay for a definitive diagnosis of TSC, followed by those with epilepsy, brain tumor/intraventricular tumor, and intellectual disabilities. The TSC clinic played an important role in the early diagnosis of TSC.

**Supplementary Information:**

The online version contains supplementary material available at 10.1186/s13023-024-03460-y.

## Background

Tuberous sclerosis complex (TSC) is a rare autosomal dominant genetic disorder caused by genetic variants in either the *TSC1* or *TSC2* gene, which encode hamartin and tuberin, respectively [1, 2]. Globally, the incidence rate of TSC is 1 in 6000–10,000 live births, and the population prevalence is 1 in 20,000 people [2–6]. According to a population survey conducted in 1997, the prevalence of TSC in Japan was 1 in 10,300 live births, which is close to global estimates [7]. TSC is characterized by the formation of hamartomas or benign tumors in various organ systems including the brain, heart, skin, lungs, and kidneys, leading to different manifestations in these organs [2]. Epilepsy and neuropsychiatric disorders, known as TSC-associated neuropsychiatric disorders, are also considered as TSC-related manifestations [2]. Some manifestations such as epilepsy, subependymal giant astrocytoma (SEGA), renal angiomyolipoma, and lymphangioleiomyomatosis can contribute to morbidity and mortality [8–11]. Many of these health risks can be minimized through early diagnosis of TSC, lifelong monitoring, and proactive treatment [12].

The diagnostic criteria for TSC, which were initially established in 1998 by Roach et al. and later updated in 2012, 2018, and 2021, provide guidelines on the diagnosis and management of TSC [6, 13, 14]. The guidelines recommend that TSC should be diagnosed through genetic testing or relevant clinical criteria. Genetic testing results indicative of pathogenic variants in *TSC1* or *TSC2* aid in TSC diagnosis, although 10–25% of patients meeting the clinical diagnostic criteria do not have any identifiable variants [6, 15]. On the other hand, the clinical diagnostic criteria comprise of 11 major and 7 minor clinical features, and a definitive TSC diagnosis can be made if the patient presents with either 2 major features or 1 major feature with 2 minor features. However, patients with TSC do not tend to meet the clinical criteria at a younger age because the peak age of onset of each manifestation in the clinical features is different in the fetal and adult phases.

Cardiac rhabdomyomas commonly occur in 40–60% of patients with TSC aged below 2 years, wherein the condition develops prenatally and can be suspected before birth in many cases [10]. In the juvenile population (< 10 years old), most (up to 80%) patients present with epilepsy and can have a diagnosis of TSC, with intracranial lesions detected through brain imaging examination at an early stage. However, the incidence of epilepsy reduces with age [10, 16]. Skin lesions such as facial angiofibroma, renal angiomyolipoma, and pulmonary manifestations such as lymphangioleiomyomatosis increase after adolescence [10]. To precisely diagnose TSC in a timely manner, understanding the time course of the manifestations and differentiating the TSC-specific manifestations from other symptoms are important.

TSC-related manifestations may not be pathognomonic or may be non-specific in the early stages [17]; therefore, if the clinician who treats the early-onset manifestations does not notify about the manifestations in other organs, then the diagnosis of TSC is presumably delayed. A multidisciplinary team approach is essential for the early diagnosis of TSC and the detection of associated lesions. Such multidisciplinary expert teams, referred to as “TSC clinic,” have been established for TSC care. In Japan, the first TSC clinic was set up at Osaka University Hospital in 2012, comprising of clinicians from different specialties (notably, pediatrics, urology, respiratory medicine, dermatology, and radiology) [18]. This multidisciplinary team holds examination days for patients once a week and consult each other on a monthly basis to determine patients’ therapeutic plans. The TSC clinics have since been replicated at other sites across Japan. The structure of the TSC clinic varies between facilities, and typically, physicians with expertise in TSC, often specialists from fields such as pediatrics or urology, lead the clinic. The physicians manage the initial care for patients with TSC, who come to the hospital and provide consultation for TSC cases discovered in other departments [19]. Although problems related to the diagnostic process of TSC and the benefits of the TSC clinic have been discussed, no studies have provided real-world data on the diagnostic flow of TSC and the effectiveness of the TSC clinic for the diagnosis of TSC in Japan. Clarifying these data, as well as following the management of each organ manifestations, presumably contribute to improve diagnostic accuracy.

This study aimed to explore the diagnostic flow of patients with TSC in the real-world setting and investigate the effect of the TSC clinic on facilitating the diagnosis. This retrospective observational study utilized data from a nationwide insurance claims database in Japan.

## Methods

### Data collection

This retrospective observational study used data from the JMDC Claims Database from January 2005 to December 2020. The database is based on health insurance claims of corporate employees and their families and is strictly anonymized and unlinkable to personal data to protect privacy. The database contains data of approximately 14 million insured persons (as of February 2022), which accounts for ~ 10% of the total population of Japan [20].

All patients with TSC had to be enrolled in health insurance at least once with at least 1 confirmed diagnosis of TSC (International Classification of Diseases, 10th edition [ICD-10] code: Q85.1) between January 2005 and December 2020 (observation period). We collected data of patient characteristics (age and gender), symptom name, disease name, date of diagnosis, types and date of examinations performed (computed tomography, magnetic resonance imaging, respiratory test, and genetic testing), and hospital types, namely university hospital, that is, a hospital affiliated with medical schools at a national, public, or private university; public hospital, that is, a national, prefectural, or municipal hospital with ≥ 20 beds; private hospital, that is, a hospital with ≥ 20 beds that cannot be categorized as a university hospital or a public hospital; and small hospital, that is, a hospital with ≤ 19 beds that are classified as a small-scaled medical facility primarily focused on outpatient care under Japan’s healthcare system. The JMDC Claims Database standardizes symptom and disease names listed on receipts (standard disease names) and links them to the ICD-10 classification codes. Disease name was extracted based on ICD-10 codes or standard disease names (Supplementary Tables 1 and 2).

The facilities that have the TSC clinic were identified in the Japanese Society of Tuberous Sclerosis Complex homepage [21]. The “month of diagnosis with TSC” was defined as the month when the patient first received a diagnosis of TSC during the observation period. The “pre-diagnosis period” was defined as the period from the start of enrollment to up to but not including the month of diagnosis with TSC. The “follow-up period” was defined as the period from the diagnosis period to the end of enrollment (excluding the month of diagnosis with TSC). We also collected data of controls (patients without a TSC diagnosis).

In this study, we classified the manifestations as follows:

1. “*TSC-related manifestations*” were defined as manifestations that specifically or frequently occur in TSC. These manifestations are further categorized as TSC-specific and non-specific TSC-related manifestations, described as follows.
2. “*TSC-specific manifestations*” comprise those included in the major and minor features in the TSC diagnostic criteria [6, 13, 14]. Due to their specificity, these manifestations are likely to lead to the diagnosis of TSC. The corresponding standard disease names are presented in Supplementary Table 1. Sclerotic bone lesion, which was newly included in the diagnostic criteria in 2021 [13], was not included.
3. “*Non-specific TSC-related manifestations*” are TSC-related symptoms other than TSC-specific manifestations. These include conditions such as epilepsy, autism spectrum disorder, and renal tumors. While these manifestations frequently occur in patients with TSC, they are not specific enough to directly lead to the diagnosis of TSC. The corresponding standard disease names are listed in Supplementary Table 2.

### Study cohorts

We initially reviewed the collected data and found that most patients under 2 years of age generally had a primary manifestation of cardiac rhabdomyoma or epilepsy and a generally shorter time to definitive diagnosis of TSC from the diagnoses of the manifestations. By contrast, patients who were diagnosed with TSC after 2 years of age may present with manifestations in various organs and require the multidisciplinary approach for the definitive diagnosis compared with patients under 2 years of age. We hypothesized that the diagnostic flow differs by patients’ age (before and after 2 years of age) and, thus, allocated our analysis into 2 cohorts (2.2.1 and 2.2.2) based on the age of TSC diagnosis.

#### Cohort 1 (age at diagnosis: ≥ 2 years)

Cohort 1 included patients whose age at TSC diagnosis was after 2 years and who have been enrolled in health insurance in the 12 months before the diagnosis.

The primary objective in this cohort was to understand the diagnostic flow of patients by examining the time to first diagnosis from each TSC-related manifestation to TSC diagnosis (time to diagnosis [TTD]).

“TTD” was calculated as months from the time of the first diagnosis for each TSC-related manifestation to the time of TSC diagnosis. In case a TSC-related manifestation was observed in the same month as that of TSC diagnosis, it was counted as 1 month.

The secondary objective was to investigate the incidence of each manifestation before TSC diagnosis in Cohort 1. The reasons for delayed diagnosis were exploratorily investigated by examining individual patient flow and testing up to TSC diagnosis as well as the facility type in which the diagnosis was made (stratified by number of beds and presence of a TSC clinic). “Delayed diagnosis” was expediently defined as more than 12 months of TTD.

#### Cohort 2 (age at diagnosis: < 2 years)

Cohort 2 included patients whose age at diagnosis was before 2 years at the time of first diagnosis and who have been enrolled in health insurance since birth (enabling follow-up since birth). The primary objective in this cohort was to understand the diagnostic flow of patients by examining TTD in patients aged < 2 years. The secondary objective was to identify TSC-specific manifestations other than known TSC-related manifestations by examining the incidence of all manifestations, which were not TSC-related manifestations before TSC diagnosis in a population of patients aged < 2 years at the time of TSC diagnosis.

We also collected data from a control group comprising patients who could be followed up from the year and month of birth and who did not have a TSC diagnosis during the observation period; the data were collected to compare the incidence of all diseases other than known TSC-related manifestations before TSC diagnosis and the risk ratio of the incidence with those of Cohort 2 patients.

### Statistical analysis

As this was a descriptive epidemiological study, endpoints were reported using summary statistics, with categorical variables presented as counts and percentages and continuous variables presented as mean, standard deviation, minimum, maximum, and median values. The person-year method (per 1000 person-months) was used to calculate the incidence rate and 95% confidence interval (CI) for the risk ratio; the 95% CI was calculated assuming an approximation of the Poisson distribution. Statistical significances for the TTD with or without TSC clinic consultation was calculated using Mann–Whitney U test. Statistical analyses were conducted using SAS for Windows 9.4 software (SAS Institute, Cary, NC, USA).

### Ethics statement

This study does not contain identifiable patient information and was exempted from medical research ethics review based on the decision of the Institutional Ethics Committee of Tottori University Hospital.

## Results

**Fig. 1:**
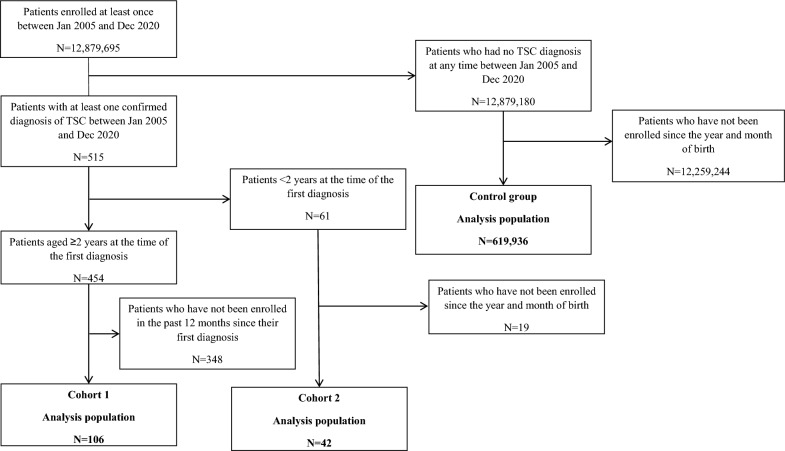
Flowchart for patient disposition

Of all patients enrolled in the JMDC database at least once during the observation period (n = 12,879,695), 106 were included in Cohort 1 and 42 were included in Cohort 2 (Fig. 1). The control group comprised 619,936 patients.

### Cohort 1 (age at diagnosis: ≥ 2 years)

#### Patient profile

Age at TSC diagnosis for Cohort 1 patients ranged from 2 to 67 years (median: 34.0 years); 25 patients were pediatric (age range: 2–18 years). Patients were observed for 16–192 months (median: 66.5 months). Most patients were diagnosed with TSC at a large facility (≥ 20 beds, 84.0%), but relatively few patients were diagnosed at a facility with a TSC clinic (33.0%; Table 1).

**Table 1:** Patient profiles in Cohort 1 (n = 106)

Profiles	Data results
Age at TSC diagnosis (years), median (range)	34.0 (2–67)
Gender, female, n (%)	63 (59.4)
Observation period (months), median (range)	66.5 (16–192)
Pre-diagnosis period, (months), median (range)	30.0 (13–182)
Facility at the time of TSC diagnosis*	
Number of beds ≥ 20, n (%)	89 (84.0)
TSC clinic, n (%)	35 (33.0)
Hospital types, n (%)	
Public hospital (other than university hospital)	12 (11.3)
University hospital	42 (39.6)
Private hospital	35 (33.0)
Small hospital (< 20 beds)	17 (16.0)

*Number of patients (percentage) who visited each facility category. TSC, tuberous sclerosis complex

### Incidence of TSC-related manifestations and time to TSC diagnosis

Of the 106 patients included in Cohort 1, 60 (56.6%) had TSC-related manifestations listed in the JMDC database before the TSC diagnosis. In Cohort 1, the symptom with the highest incidence was epilepsy (29.2%), followed by renal tumor (9.4%) and brain tumor/intraventricular tumor (8.5%); there were no reports of subependymal nodules (SEN) or SEGA (Table 2).

**Table 2:** Incidence and age at TSC diagnosis of symptoms/disease and TTDs in Cohort 1 (N = 106)

TSC-related manifestations	Incidence	Age at TSC diagnosis (years)		TTD (months)	
	n (%)	Median	Range	Median	Range
TSC-specific manifestations	23 (21.7)	34	2.0–58	1	1–27
*Skin condition*					
Facial angiofibroma	8 (7.5)	19	1.0–43	1	1–3
Shagreen patch	1 (0.9)	40	–	16	16–16
Hypomelanotic macules	1 (0.9)	13	1.0–13	1	1–1
*Cardiac symptoms*					
Cardiac rhabdomyoma	6 (5.7)	13	2.0–47	9.5	1–78
*Renal symptoms*					
Renal angiomyolipoma	6 (5.7)	40	2.7–58	4	1–21
*Pulmonary symptoms*					
Lymphangioleiomyomatosis	7 (6.6)	39	1.7–51	1	1–12
Non-specific TSC-related manifestations	45 (42.5)	34	2.0–61	11	1–84
*Neurological disorder*					
Brain tumor/intraventricular tumor	9 (8.5)	28	3.6–56	14	1–84
Epilepsy	31 (29.2)	30	2.6–61	14	1–89
Intellectual disability	5 (4.7)	20	6.0–37	12	1–68
Developmental disorder	4 (3.8)	17.5	1.5–31	1	1–3
*Renal symptoms*					
Renal tumor	10 (9.4)	38.5	5.2–58	23	1–91
Malignant renal tumor	5 (4.7)	46	1.0–56	2	1–32
*Other organ symptoms*					
Ovarian cyst	3 (2.8)	26	6.0–34	16	8–23

TSC, tuberous sclerosis complex; TTD, time to diagnosis, defined as months from the time to first diagnosis for each TSC-related manifestation to the time of TSC diagnosis; SD, standard deviation; SEGA, subependymal giant cell astrocytoma; SEN, subependymal nodule

SEN, SEGA, or cortical tuber was not recorded in the cohort data

**Fig. 2:**
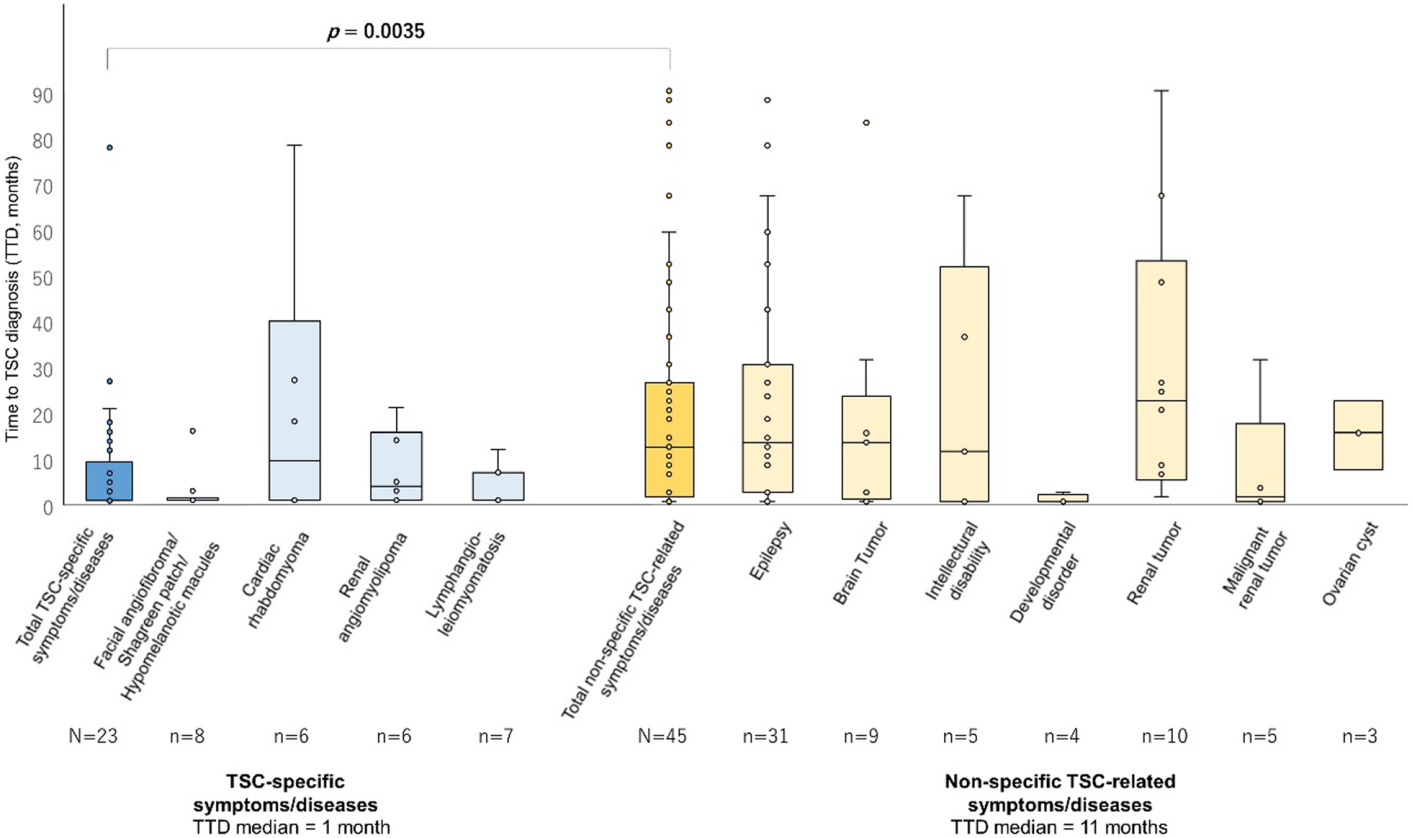
Time to TSC diagnosis (TTD) by each TSC-related manifestation

In the TTD analysis, TTDs for TSC-specific manifestations (median: 1 month, range: 1–27 months) were significantly shorter than those for non-specific TSC-related manifestations (median: 11 months, range: 1–84 months; *p* = 0.0035; Fig. 2, Table 2). Among the TSC-specific manifestations, TTDs for facial angiofibroma, hypomelanotic macules, and lymphangioleiomyomatosis were short (median: 1 month; Table 2).

The boxes indicate interquartile range with median. Whiskers mean the maximum and minimum values excluding outliers. Facial angiofibroma, Shagreen patch, and hypomelanotic macules were each seen in a small number of patients and were included in skin manifestations. A statistically significant difference among TTDs in TSC-specific and non-specific TSC-related manifestations was revealed when tested with Mann–Whitney U test.

TSC, tuberous sclerosis complex; TTD, time to diagnosis, defined as months from the timing of diagnosis for each TSC-related manifestation to the timing of TSC diagnosis.

### Impact of TSC clinic consultation on TTD

Table 3 shows data of the occurrence of TSC-related manifestations and their TTDs in patient groups who visited facilities with a TSC clinic (TSC clinic group) and those without a TSC clinic (non-TSC clinic group) during the pre-diagnosis period. The TTDs of all manifestations were not statistically significantly different between the TSC clinic group (median: 3.0 months, range: 1–49 months) and the non-TSC clinic group (median: 13.0 months, range: 1–91 months). However, in the analysis for each manifestation, TTD of epilepsy was significantly shorter in the TSC clinic group (median: 11.5 months, range: 1–31 months) than in the non-TSC clinic group (median: 19.0 months, range: 1–89 months). By contrast, TTDs of other manifestations were not significantly different among the groups. However, the longest TTDs in each manifestation were seen in the non-TSC clinic group in all manifestation categories other than malignant renal tumor.

**Table 3:** TTDs of TSC-related manifestations in association with patient groups in Cohort 1 during the pre-diagnosis period

TSC-related manifestations	TSC clinic group (n = 25)		Non-TSC clinic group (n = 35)		Comparison of TTDs (*p*-value)
	n (%)	TTDs (months) median (range)	n (%)	TTDs (months) median (range)	
All manifestations	25 (41.7)	3.0 (1–49)	35 (58.3)	13.0 (1–91)	0.0966
Brain tumor/intraventricular tumor	2 (3.3)	23.0 (14–32)	7 (11.7)	3.0 (1–84)	0.4623
Epilepsy	12 (20.0)	11.5 (1–31)	19 (31.7)	19.0 (1–89)	0.0379*
Intellectual disability	2 (3.3)	6.5 (1–12)	3 (5.0)	37.0 (1–68)	0.5536
Cardiac rhabdomyoma	2 (3.3)	1.0 (1–1)	4 (6.7)	22.5 (1–78)	0.2188
Total renal manifestations	8 (13.3)	17.5 (1–49)	10 (16.7)	8.0 (1–91)	0.9289
Renal angiomyolipoma	2 (3.3)	8.5 (3–14)	4 (6.7)	3.0 (1–21)	0.8143
Renal tumor	4 (6.7)	23.0 (2–49)	6 (10.0)	18.0 (2–91)	0.8307
Malignant renal tumor	3 (5.0)	2.0 (1–32)	2 (3.3)	2.5 (1–4)	1

m, month(s); TSC, tuberous sclerosis complex; TTD, time to diagnosis, defined as months from the timing of diagnosis for each TSC-related manifestation to the timing of TSC diagnosis. The groups of TSC clinic/non-TSC clinic, respectively, include patients who visited facilities with/without a TSC clinic during the pre-diagnosis period. For the comparisons, Mann–Whiteney U test was used. *: *p* < 0.05

### Patient examples with long TTD regarding non-specific TSC-related manifestations

Supplementary Figure shows cases with long TTDs (range: 49–91 months) regarding non-specific TSC-related manifestations including brain tumor, epilepsy, renal tumor, and intellectual disability. Three patients (Patients 1, 3, and 4) were diagnosed with TSC withing 2 months after the diagnoses of TSC-specific manifestations including hypomelanotic macules, renal angiomyolipoma, and cardiac rhabdomyoma. Two patients (Patients 2 and 4) were diagnosed with TSC after transfer to the hospital with a TSC clinic, where the diagnosis of renal tumor was corrected to renal angiomyolipoma (Patient 4).

### Cohort 2 (age at diagnosis: < 2 years)

#### Patient profiles

Age at diagnosis for TSC in Cohort 2 ranged between 1 and 20 months (median: 5 months). Cardiac rhabdomyoma (54.8%) and epilepsy (38.1%) were major symptoms that manifested before TSC diagnosis (Table 4).

**Table 4:** Patient profiles in Cohort 2 (n = 42)

Profiles	Data results
Age at TSC diagnosis (months), median (range)	5 (1–20)
Gender, male, n (%)	26 (61.9)
Facility types at the time of TSC diagnosis*	
Number of beds, ≥ 20, n (%)	41 (97.6)
With TSC clinic, no, n (%)	23 (54.8)
*Hospital types, n (%)*	
Public hospital	20 (47.6)
University hospital	13 (31.0)
Private hospital	8 (19.0)
Clinic (< 20 beds)	1 (2.4)
*Manifestations before TSC diagnosis*	
Neurological manifestations, n (%)	
Brain tumor	4 (9.6)
Epilepsy	16 (38.1)
Developmental disorder	2 (4.8)
*Skin manifestations, n (%)*	
Hypomelanotic macules	5 (11.9)
*Cardiac manifestations, n (%)*	
Cardiac rhabdomyoma	23 (54.8)
*Renal manifestations, n (%)*	
Renal tumor	1 (2.4)
Renal cyst	1 (2.4)

*Number of patients (percentage) who visited each facility type

TSC, tuberous sclerosis complex

### Incidence of all manifestations other than TSC-related manifestations and the occurrence risks compared with control group patients

We collected the data of 619,926 control group patients from the JMDC Claims Database. Manifestations with a risk ratio of ≥ 1.5 and 95% CI with a lower bound greater than 1.0 are listed in Table 5. The most common manifestation in patients with TSC was dry skin, which occurred in 27 patients (64.3%; incidence rate: 52.6 per 1000 person-months) in Cohort 2, and with an incidence risk ratio of 1.7 compared with that of control group patients.

**Table 5:** Incidence of manifestations in patients of Cohort 2 with a risk ratio of ≥ 1.5 compared with the control group

Standard disease name	Incidence n (%)		Incidence rate (/1000 person-months)		Risk ratio	95% CI
	Cohort 2 (N = 42)	Control group (N = 619,936)	Cohort 2 (N = 42)	Control group (N = 619,936)		
Dry skin	27 (64.3)	271,315 (43.8)	52.6	31.2	1.7	1.16–2.46
Vomiting	13 (31.0)	86,663 (14.0)	16.8	7.6	2.2	1.29–3.81
Dehydration	12 (28.6)	53,998 (8.7)	17	4.6	3.7	2.09–6.49
Pyrexia	12 (28.6)	93,051 (15.0)	16.6	8.2	2	1.14–3.55
Vitamin K deficiency neonatal	11 (26.2)	21,276 (3.4)	16.2	1.8	9.1	5.02–16.36
Hand, foot, and mouth disease	10 (23.6)	73,205 (11.8)	12.9	6.3	2.1	1.11–3.83
Transient hyperpnea of the newborn	8 (19.0)	27,289 (4.4)	11	2.3	4.7	2.36–9.45
Iron-deficiency anemia	8 (19.0)	25,944 (4.2)	10.5	2.2	4.8	2.40–9.60
Febrile convulsion	8 (19.0)	23,875 (3.9)	10.5	2	5.3	2.65–10.62
Vitamin B6 deficiency	7 (16.7)	163 (0.0)	9.4	0	704.8	330.73–1501.78
Hyperopic astigmatism	7 (16.7)	5580 (0.9)	9.1	0.5	20.0	9.52–41.91
Hamartoma	6 (14.3)	14 (0.0)	7.9	0	6886.5	2646.35–17,920.49

CI, confidence interval

The risk ratios were obtained by comparing the data of Cohort 2 with those of the control group

Deficiency of vitamin K (risk ratio: 9.1) and vitamin B6 (704.8), hyperopic astigmatism (20.0), and hamartoma (6886.5), which were unrelated to TSC, were also manifestations with a high risk ratio.

## Discussion

This is the first retrospective observational study based on a nationwide health insurance claims database to explore the real-world diagnostic flow and TTD of TSC in Japan. We analyzed the data of patients who were diagnosed with TSC after 2 years (mostly in adulthood) in Cohort 1. From this cohort, re-evaluation of TSC-related manifestations might lead to TSC diagnosis in adulthood. The system of the TSC clinic was considered useful for reducing the time to TSC diagnosis when epilepsy, among TSC-related manifestations, appeared in patients. Based on the analysis of Cohort 2, which assessed patients whose age at diagnosis was under 2 years, it is presumably important for early TSC diagnosis before the age of 2 years to link the cardiac rhabdomyomas and epilepsy to the evaluating manifestations of TSC.

### Cohort 1

#### Associations between TTD and onset timing of each manifestation

In Cohort 1, we analyzed the data of patients with a TSC diagnosis after the age of 2 years, and the results showed that the age at diagnosis was distributed majorly in adulthood (median: 34.0 years). Epilepsy showed the highest incidence among the TSC-related manifestations, followed by renal tumor and brain tumor. Non-specific TSC-related manifestations such as renal tumor and neurological disorders (brain tumor/intraventricular tumor, epilepsy, and intellectual disability) were associated with a long TTD, and renal tumor, particularly, required the longest time to TSC diagnosis (median: 23 months, range: 2–91 months). By contrast, patients with TSC-specific manifestations of renal angiomyolipoma and cardiac rhabdomyoma (which had a lower incidence of 5.7% each) as the first manifestation had a relatively shorter TTD (median: 4.0 and 9.5 months, respectively).

In this study, non-specific TSC-related manifestations may have been identified without recognizing that TSC was an underlying condition, resulting in the delay of TSC diagnosis. In patients who finally had a definitive diagnosis with TSC, renal angiomyolipoma might have been incorrectly diagnosed as “renal tumor” or “malignant renal tumor,” and SEN/SEGA or cortical tuber was possibly misdiagnosed as “brain tumor” owing to the physicians’ lack of knowledge and experience on TSC diagnosis. The short TTDs for TSC-specific manifestations presumably indicate that the physician who could notify those specific findings and/or diagnosed the patients as TSC at the timings. The TSC-specific manifestations observed in Cohort 1, including facial angiofibroma, Shagreen patch, hypomelanotic macules, cardiac rhabdomyoma, renal angiomyolipoma, and lung lymphangioleiomyomatosis, are determining findings leading to the diagnosis of TSC and can be the key findings of the diagnosis in the late phases of life. Dispersing the knowledge about these TSC symptoms/disease findings to the physicians potentially treating adult patients may help achieve the diagnosis of TSC in overlooked cases.

Interestingly, no patients were diagnosed with TSC based on the TSC-specific neurological manifestations of SEN or SEGA after 2 years of age. The incidence of SEGA has been reported to be 26% for patients aged ≤ 18 years and 14% for older patients; thus, circumstantially, there may have been no patients with symptomatic SEGA in Cohort 1, where most of the patients had a diagnosis in adulthood. Alternatively, patients with SEGA might have been diagnosed only with a brain tumor. SEN is a finding observed in most patients with TSC [6]; however, it might be listed as an intraventricular tumor or not be listed as a diagnosis due to the absence of a directly corresponding standard disease name for insurance claims and its limited clinical significance.

### Associations between TSC clinic consultation and TTD

Our analyses also showed that the patients attending facilities with a TSC clinic were diagnosed with TSC more quickly than those attending facilities without a TSC clinic when the patients develop epilepsy. Although not statistically significant, even in the overall analysis of TSC-related manifestations, patients attending facilities with a TSC clinic (median: 3.0 months) tended to have a shorter time to TSC diagnosis compared with those attending facilities without a TSC clinic (13.0 months). The lack of a multidisciplinary care system comprising TSC experts in facilities without a TSC clinic could be one reason for the observed delay in TSC diagnosis. A multidisciplinary approach with the involvement of specialists, if feasible, is recommended by the updated TSC guidelines to facilitate diagnosis [13]. This recommendation is supported by single-center experiences from France and Japan, wherein a multidisciplinary model was shown to provide optimal TSC patient care [19, 22]. Fujimoto et al. clearly showed an improvement in patient care as well as earlier diagnosis of TSC upon the establishment of a TSC clinic in Japan [19]. Our data support the advantage of a TSC clinic based on the large-scale database.

Great importance is placed on the need for patients to access clinicians with expertise in TSC, as it is a complex and multisystem disease, often involving non-specific manifestations that may confound diagnosis made by non-expert clinicians [23]. In many of the patients with TSC who did not visit a hospital with a TSC clinic, as in the case of Patients 2 and 3 (Supplementary Figure), the TTD for TSC was longer despite a combination of manifestations, which can lead to TSC diagnosis. As shown in Patient 4, the diagnosis of a renal tumor was changed to renal angiomyolipoma after visit to a hospital with a TSC clinic. It was considered that internal cooperation between specialists in the TSC clinic could prevent confounding diagnosis, resulting in earlier diagnosis. Hence, facilitation of patient referral to a hospital with a TSC clinic may contribute to early diagnosis of TSC.

### Cohort 2

#### Associations between TTD and onset timing of each manifestation

In Cohort 2, patients who had a TSC diagnosis before 2 years of age mainly presented with epilepsy or cardiac rhabdomyoma. Cardiac rhabdomyomas are an early manifestation of TSC in newborns, with the occurrence of multiple rhabdomyomas being significantly associated with TSC [3, 24], which tend to regress spontaneously in most cases [25]. Furthermore, 70–90% of newborns with cardiac rhabdomyoma have TSC [26]. Cardiac rhabdomyomas are also key findings for the early diagnosis of TSC in patients with hypomelanotic macules [27]. Therefore, infants in whom cardiac rhabdomyomas are detected would be screened for TSC. Epilepsy also has a high incidence rate in patients, with some patients developing clinical seizures soon after birth [8]. The onset of seizures tends to be the first symptom, which serve as a trigger for medical attention and the identification of brain lesions, cardiac rhabdomyomas, and/or hypomelanotic macules leading to the diagnosis of TSC [27]. However, as seen in patients of Cohort 1, the brain lesions did not lead to the diagnosis of TSC in some patients. Patients with TSC who developed epilepsy early tend to have severe intracranial lesions, infantile epileptic spasms syndrome, and severe neurological outcomes [28, 29]. Vigabatrin is the key treatment for patients with TSC developing infantile epileptic spasms syndrome [30]. Dispersing knowledge about the characteristics of the brain lesions in TSC to pediatricians and secured diagnosis of TSC by prompt neuroimaging is important for early diagnosis and appropriate treatment.

### Associations between TSC and other manifestations or conditions

We further reviewed the data to determine the presence of any other manifestations, which are closely associated with TSC, to enable clinicians to suspect a diagnosis of TSC early in life. Dry skin was the most common non–TSC-related symptom among patients under 2 years of age at the time of TSC diagnosis. Although various factors are related to dry skin, activation of the mammalian target of rapamycin pathway is one possible mechanism associated with dry skin in TSC [31]. Dry skin is quite a common symptom in the general population, and it may be difficult to associate it with TSC diagnosis for physicians. Hamartomas, such as cortical tubers, retinal hamartomas, and other tumor lesions, were presumed to have been diagnosed in patients with TSC, leading to a high incidence rate. However, it was not possible to identify which organ-specific lesions the diagnosed hamartomas referred to from the database. Deficiency of vitamin K and vitamin B6 and hyperopic astigmatism were also manifestations with a higher incidence rate in the TSC cohort than in the control group. However, these diagnoses are considered not to be directly caused by the pathophysiology of TSC. High-dose vitamin B6 is a treatment option for infantile epileptic spasms syndrome, which frequently occurs in TSC [32, 33]; vitamin K is administered for all patients hospitalized during the neonatal period. Thus, the diagnosis of vitamin B6 and K deficiency might be arbitrarily used for health insurance claims. Hyperopic astigmatism is also conveniently diagnosed for a descriptive purpose when a patient undergoes ophthalmological examinations in Japan.

### Limitations

The study limitations are largely related to the nature of the claims database. As this study is based on the database that covered only people aged < 74 years who were members of a health insurance association, the results may not be generalizable to the entire Japanese population. The same patient might have been included twice if the patient had changed insurance policies and joined a different health insurance association. Although the names of the diagnosis were based on the diseases listed on the insurance claim, it may not necessarily represent the actual health condition of the patient, as it may have been listed just to prescribe the associated drug or conduct each examination. It was considered that TSC-specific diagnoses are more likely to be made by physicians at TSC multidisciplinary teams, but this trend could not be investigated in the current study. In Cohort 1, it is not definitive that TSC was diagnosed at the first time for the patient because the diagnostic data from birth were untraceable as shown in the inclusion criteria for Cohort 1. We could not consider the timing of the imaging study because the body sites of image scanning were not specified in the claims database. In this study, the number of lesions could not be captured from the database, although information of the number of lesions was needed for facial angiofibroma, hypomelanotic macules, and renal angiomyolipoma to meet the TSC diagnostic criteria. In addition, hypomelanotic macules could not be differentiated from vitiligo in Japanese and were hence recorded as vitiligo because hypomelanotic macules do not have an ICD-10 code. Several TSC-associated features such as periungual fibrosis and SEN were not recorded in the database, as they do not require treatment.

## Conclusions

Our real-world analysis of TSC patient flow showed severe delay in TSC diagnosis in some cases of non-specific TSC-related manifestations such as renal tumor, brain tumor, or epilepsy as a primary symptom manifestation. The manifestations might have been misdiagnosed or not have been associated with TSC due to the physicians’ lack of knowledge and experience on TSC diagnosis. The TSC multidisciplinary team played an important role in the early diagnosis of TSC, as specialists in TSC clinic can suspect TSC involving non-specific TSC-related manifestations.

### **EQUATOR Reporting guidelines**:

STROBE: Guidance on reporting observational studies in epidemiology (i.e. RWE). RECORD: Guidance on reporting observational studies using routinely collected health data.

## Supplementary Information

Additional file 1

Additional file 2

Additional file 3

## Data Availability

The datasets used and/or analyzed during this study are not publicly available but can be requested from the corresponding author upon reasonable request.

## Abbreviations

CI: Confidence interval
ICD-10: International classification of diseases, 10th edition
SEGA: Subependymal giant astrocytoma
SEN: Subependymal nodules
TTD: Time to diagnosis
TSC: Tuberous sclerosis complex
