# Supplementary material for: Diagnostic flow analysis of tuberous sclerosis complex in Japan: a retrospective claims database study
Source: Orphanet J Rare Dis. 2024 Dec 1;19:451. doi: 10.1186/s13023-024-03460-y (PMC11610214; doi:10.1186/s13023-024-03460-y)
Supplement: Supplementary file 1 — Additional file 1 [file 13023_2024_3460_MOESM1_ESM.pptx]

## Slide 1
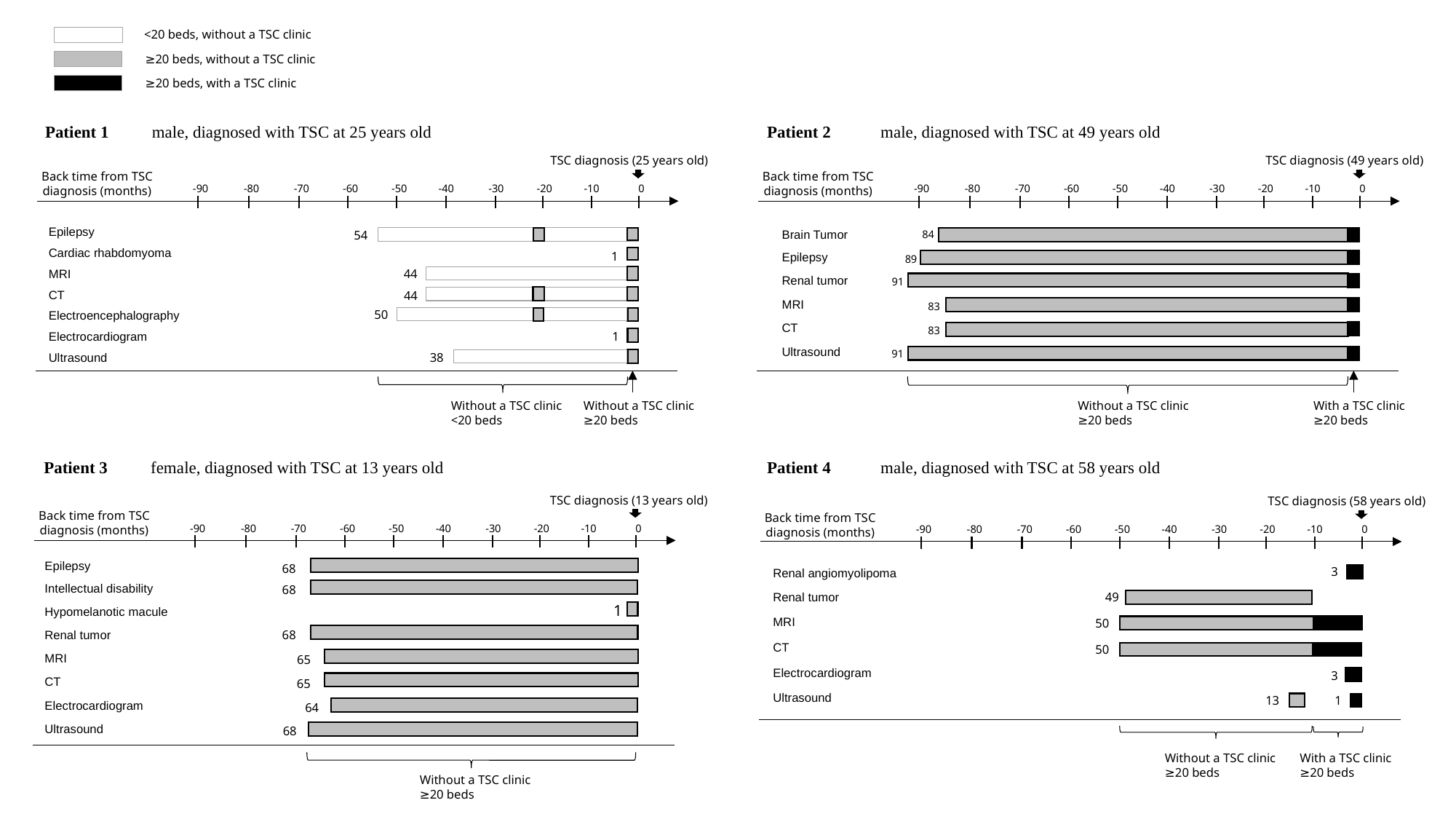

<20 beds, without a TSC clinic
≥20 beds, without a TSC clinic
≥20 beds, with a TSC clinic
Patient 1
male, diagnosed with TSC at 25 years old
Patient 2
male, diagnosed with TSC at 49 years old
TSC diagnosis (25 years old)
Back time from TSC diagnosis (months)
-90
-80
-70
-60
-50
-40
-30
-20
-10
0
54
1
44
44
50
1
38
Without a TSC clinic
<20 beds
Without a TSC clinic
≥20 beds
TSC diagnosis (49 years old)
Back time from TSC diagnosis (months)
-90
-80
-70
-60
-50
-40
-30
-20
-10
0
Without a TSC clinic
≥20 beds
With a TSC clinic
≥20 beds
| Epilepsy |
| --- |
| Cardiac rhabdomyoma |
| MRI |
| CT |
| Electroencephalography |
| Electrocardiogram |
| Ultrasound |
| Brain Tumor |
| --- |
| Epilepsy |
| Renal tumor |
| MRI |
| CT |
| Ultrasound |
84
89
91
83
83
91
Patient 3
female, diagnosed with TSC at 13 years old
Patient 4
male, diagnosed with TSC at 58 years old
TSC diagnosis (13 years old)
TSC diagnosis (58 years old)
Back time from TSC diagnosis (months)
-80
-70
-60
-50
-40
-30
-20
-10
0
-90
Back time from TSC diagnosis (months)
-90
-80
-70
-60
-50
-40
-30
-20
-10
0
| Epilepsy |
| --- |
| Intellectual disability |
| Hypomelanotic macule |
| Renal tumor |
| MRI |
| CT |
| Electrocardiogram |
| Ultrasound |
68
3
| Renal angiomyolipoma |
| --- |
| Renal tumor |
| MRI |
| CT |
| Electrocardiogram |
| Ultrasound |
68
49
1
50
68
50
65
3
65
13
1
64
68
Without a TSC clinic
≥20 beds
With a TSC clinic
≥20 beds
Without a TSC clinic
≥20 beds
